# Penumbra Pattern Assessment in Acute Stroke Patients: Comparison of Quantitative and Non-Quantitative Methods in Whole Brain CT Perfusion

**DOI:** 10.1371/journal.pone.0105413

**Published:** 2014-08-21

**Authors:** Kolja M. Thierfelder, Louisa von Baumgarten, Alena B. Baumann, Felix G. Meinel, Andreas D. Helck, Christian Opherk, Andreas Straube, Maximilian F. Reiser, Wieland H. Sommer

**Affiliations:** 1 Department of Clinical Radiology, Ludwig-Maximilians-University of Munich Hospitals, Munich, Germany; 2 Department of Neurology, Ludwig-Maximilians-University of Munich Hospitals, Munich, Germany; 3 Institute for Stroke and Dementia Research, Ludwig-Maximilians-University of Munich Hospitals, Munich, Germany; University Medical Center (UMC) Utrecht, Netherlands

## Abstract

**Background And Purpose:**

While penumbra assessment has become an important part of the clinical decision making for acute stroke patients, there is a lack of studies measuring the reliability and reproducibility of defined assessment techniques in the clinical setting. Our aim was to determine reliability and reproducibility of different types of three-dimensional penumbra assessment methods in stroke patients who underwent whole brain CT perfusion imaging (WB-CTP).

**Materials And Methods:**

We included 29 patients with a confirmed MCA infarction who underwent initial WB-CTP with a scan coverage of 100 mm in the z-axis. Two blinded and experienced readers assessed the flow-volume-mismatch twice and in two quantitative ways: Performing a volumetric mismatch analysis using OsiriX imaging software (MM_VOL_) and visual estimation of mismatch (MM_EST_). Complementarily, the semiquantitative Alberta Stroke Programme Early CT Score for CT perfusion was used to define mismatch (MM_ASPECTS_). A favorable penumbral pattern was defined by a mismatch of ≥30% in combination with a cerebral blood flow deficit of ≤90 ml and an MM_ASPECTS_ score of ≥1, respectively. Inter- and intrareader agreement was determined by Kappa-values and ICCs.

**Results:**

Overall, MM_VOL_ showed considerably higher inter-/intrareader agreement (ICCs: 0.751/0.843) compared to MM_EST_ (0.292/0.749). In the subgroup of large (≥50 mL) perfusion deficits, inter- and intrareader agreement of MM_VOL_ was excellent (ICCs: 0.961/0.942), while MM_EST_ interreader agreement was poor (0.415) and intrareader agreement was good (0.919). With respect to penumbra classification, MM_VOL_ showed the highest agreement (interreader agreement: 25 agreements/4 non-agreements/κ: 0.595; intrareader agreement 27/2/0.833), followed by MM_EST_ (22/7/0.471; 23/6/0.577), and MM_ASPECTS_ (18/11/0.133; 21/8/0.340).

**Conclusion:**

The evaluated approach of volumetric mismatch assessment is superior to pure visual and ASPECTS penumbra pattern assessment in WB-CTP and helps to precisely judge the extent of 3-dimensional mismatch in acute stroke patients.

## Introduction

The selection of patients who may benefit from reperfusion therapy is a major issue in acute stroke imaging [1]. A mismatch between infarct core and ischemic tissue (penumbra) is considered as potentially savable “tissue at risk”. It has been shown that its presence and extent provides guidance for therapeutic decisions [2]. CT perfusion (CTP) mismatch assessment gains importance in clinical practice and has become a major imaging method in large clinical trials as a fast, reliable and widely available alternative to magnetic resonance imaging (MRI) [3]. Recent studies have used the extent of blood flow-blood volume mismatch in CTP or perfusion-diffusion mismatch in MRI as their inclusion criterion for reperfusion therapy [1],[4].

However, there is no consensus on the exact definition of a “substantial” or “meaningful” mismatch suggesting that there is a considerable volume of savable tissue, supporting the decision for reperfusion therapy [5],[6]. In a recent and controversially discussed publication on the patient selection for endovascular therapy, such a “favorable penumbra pattern” was defined as mismatch of ≥30% together with an infarct core of ≤90 ml [1]. Other cutoff values, e.g. a mismatch of ≥20% with no maximum infarct core, have also been used [4].

While mismatch assessment has become an important part of the clinical decision making for acute stroke patients, there is a lack of studies measuring the reliability and reproducibility of defined assessment techniques in the clinical setting. In most clinical trials, the penumbra is being visually estimated by the reader without any further, more objective quantification. MR studies on perfusion-diffusion (PWI-DWI) mismatch, however, have shown that visual estimation of mismatch has poor reliability [7]. This relates to the fact that it is challenging to visually assess 3-dimensional volume differences from axial images. As recent technical developments in CTP have led to an extension of the scan range to cover the entire brain in the z-axis, modern whole brain CTP (WB-CTP) allows to determine the total volume of the ischemic region [8]–[10]. This development reinforces doubts whether plain visual mismatch estimation in CT perfusion is still adequate. Approaches of quantitative volumetric mismatch assessment have been proposed but still need to be systematically analyzed [11]. A semiquantitative approach is the Alberta Stroke Programme Early CT Score (ASPECTS) [12]. The ASPECTS concept is also applied to describe the flow-volume mismatch on standard CTP maps (CTP-ASPECTS) [2],[13].

The objective of this study was to examine reliability and reproducibility of a quantitative volumetric mismatch assessment and to compare it to visual CBF-CBV-mismatch assessment and to CTP-ASPECTS.

## Methods

### Patient Population

The institutional review board of the Ludwig-Maximilians-University of Munich (Germany) approved the study and waived requirement for informed consent as the data was analyzed anonymously. Between January 2012 and April 2012, 30 patients with acute ischemic stroke who were referred for multimodal cranial CT including a WB-CTP scan were included in this study. Inclusion criteria were (1) complete whole brain CBF and CBV datasets, and (2) confirmed unilateral middle cerebral artery (MCA) territory ischemic stroke as confirmed by follow-up MRI. Exclusion criterion was an inadequate quality of the CTP datasets.

### Ct Examination Protocol

The CT protocol consisted of a non-enhanced CT (NECT) to exclude intracerebral hemorrhage, a supra-aortic CT angiography and a WB-CTP, all being performed using a 128-row CT scanner (*SOMATOM Definition Flash*; Siemens Healthcare, Forchheim, Germany). WB-CTP images were obtained with 0.6 mm collimation and scan coverage of 100 mm in the z-axis using a toggling table technique. 31 axial slices with a thickness of 10 mm and an increment of 3 mm were acquired continuously over 48 seconds (32 cycles, one sweep every 1.5 sec). Tube voltage and current were set to 80 kV and 200 mAs, respectively. CTDI_vol_ was 276.21 mGy. 35 mL of a highly-iodinated contrast agent were administered at a flow rate of 5 mL/s, followed by a saline flush of 40 mL at 5 mL/s.

### Image Processing

The axial CTP images were transferred to a dedicated workstation and perfusion analysis was performed with the vendor given *Syngo VPCT Neuro* software using a semi-automated deconvolution algorithm (*Auto Stroke MTT*) [8]. The arterial input function was chosen as a region of interest from the main arterial vessels on a representative slice on the level of basal ganglia. The venous output region was selected from the superior sagittal sinus. For both CBF and CBV, a set of 31 color coded slices were reconstructed. These sets were presented together with a color scale next to the respective map, allowing direct read out absolute values of the complete spectrum of the perfusion parameters. An adjustment of the window settings is not intended by the vendor.

### Image Analysis

We performed both quantitative and semiquantitative assessment of mismatch: For quantitative evaluation, all perfusion map datasets were assessed twice by each of the two readers using two different quantitative mismatch assessment methods: (a) volumetric mismatch (MM_VOL_) and (b) visually estimated mismatch (MM_EST_). We acknowledge that there is no consensus on a favorable penumbral pattern or a substantial mismatch. We defined a favorable penumbral pattern as a mismatch of ≥30% together with a CBV deficit of ≤90 ml (substantial salvageable tissue and small infarct core, all other patients were classified to have a nonpenumbral pattern (large core or small or absent penumbra). This is a definition that is closely linked to the favorable penumbra pattern of Kidwell et al [1].

The semiquantitative evaluation of the mismatch extent was performed using the Alberta Stroke Programme Early CT Score for CT perfusion (CTP-ASPECTS mismatch, MM_ASPECTS_).

All readings were performed independently by two blinded readers, each with six years of experience in CTP reading and ASPECTS. No clinical data were provided to the readers. We kept at least 2-week intervals between all readings.

#### Quantitative Mismatch Types (a): Volumetric Mismatch (mm_Vol_)

Reconstructed axial CTP images were transferred to a 27” *iMac* computer (Apple Inc., Cupertino/CA, USA). *OsiriX V.4.0* imaging software was used to calculate the total volume of the respective CBF and CBV deficits as described before [14]. For both datasets, the perfusion deficit was segmented on every third slice. We manually outlined the respective deficits using the *OsiriX* closed polygon tool, thereby creating a region of interest (ROI).

We deliberately avoided using rigid quantitative thresholds for the definition of infarct core and hypoperfused area in order to identify artifacts, discriminate between grey and white matter, and to preserve the option to individually compare to the contralateral side.

ROIs in between the segmented slices were interpolated automatically. The resulting mismatch volume was determined as follows:

#### Quantitative Mismatch Types (b): Visually Estimated (mm_Est_) Mismatch

Readers estimated the extent of the total flow-volume mismatch in 10% steps without the use of any additional quantitative tools. Mismatch was defined as stated above. In addition, readers were asked to classify the mismatch pattern in (a) a favorable penumbral pattern – estimated mismatch of ≥30% and estimated infarct core of ≤90 ml – or (b) a nonpenumbral pattern.

#### Semiquantitative Mismatch (aspects Mismatch, Mm_Aspects_)

In a separate session, all perfusion deficits were described using the Alberta Stroke Programme Early CT Score for CT-perfusion (CTP-ASPECTS) and CBF-CBV ASPECTS mismatch was determined.

**Figure 1:**
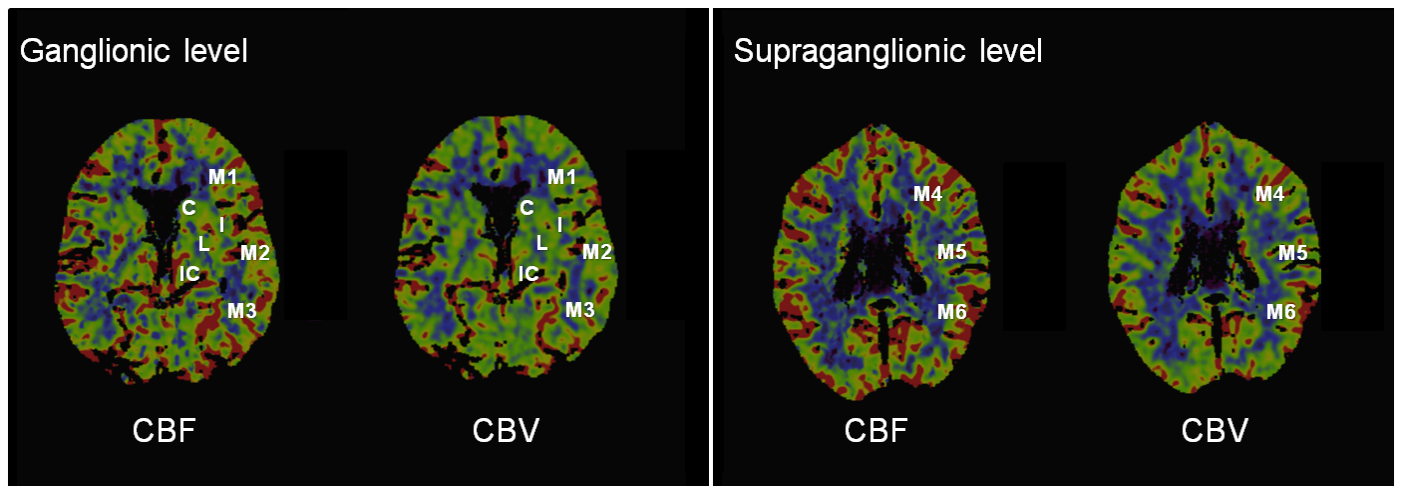
ASPECTS for mismatch assessment in CT perfusion. C, caudate head; I, insular ribbon; IC, internal capsule; L, lentiform nucleus; M1-M6, MCA region 1–6; CBF, cerebral blood flow; CBV, cerebral blood volume.

ASPECTS is a semiquantitative topographic CT score. It describes alterations of CT maps in 10 regions of the MCA territory using two axial slices (Figure 1). On the ganglionic level, the integrity of the caudate head, the lentiform nucleus, the internal capsule, the insular ribbon, the M1 (anterior MCA cortex), the M2 (MCA cortex lateral to insular ribbon), and the M3 (posterior MCA cortex) regions is assessed. On the supraganglionic level, M4, M5, and M6 as anterior, lateral and posterior MCA regions are being evaluated [12]. The number of regions with no CT irregularities is counted (maximum points 10 for a normal CT scan). The ASPECTS CT perfusion mismatch (MM_ASPECTS_) was defined as follows [13]:

MM_ASPECTS_ of ≥1 was considered a substantial mismatch [13]. According to Abels et al. [15], we chose CBF for the definition of the ischemic region as it is more specific than MTT and has a physiological correlate [16].

Both inter- and intrareader agreement was determined for all patients and additionally in 2 subgroups: patients with large (CBF≥50 mL) and small (CBF<50 mL) perfusion deficits.

### Time Measurements

The total time needed by each reader to determine for the respective mismatch assessment (MM_VOL_, MM_EST_, MM_ASPECTS_) as described above was recorded for all readings.

### Statistical Analysis

We performed all statistical analyses using SPSS Statistics 21 (IBM, Armonk/NY, USA). Normal distribution was assessed using the Kolmogorov-Smirnov test. P-values below 0.05 were considered significant. Correlation between the quantitative mismatch types was assessed by Pearson's correlation coefficient for normally distributed variables and Spearman's correlation coefficient for non-normally distributed variables. 95% confidence intervals (CI) were calculated and two-sided p-values were determined. Single measure intraclass correlation coefficients (ICCs, 2-way random) were calculated to assess and compare inter- and intrareader variabilities of the different mismatch assessment methods. The model utilized the (more strict) absolute agreement option, which measures whether the same absolute score was assigned.

Inter- and intrareader variability of the semiquantitative ASPECTS mismatch assessment as well as penumbra pattern classification was assessed by Cohen's Kappa (κ).

## Results

Out of 30 patients that were initially included, one was excluded from further analysis due to severe motion artifacts. Dose-length-product (DLP) for WB-CTP was 3257 mGy x cm, resulting in an effective radiation dose of 6.8mSv per patient. Patient characteristics are shown in Table 1.

**Table 1:** Patient characteristics (N = 29).

	Value	Range
Age (years) ±SD	71.1±12.9	34 to 93
Male gender	52%	
Perfusion deficit on right side	45%	
Time from symptom onset (min) ±SD	209±131	85 to 570
NIHSS on admission ±SD	5.8±4.6	2 to 17

NIHSS: National Institutes of Health Stroke Scale.

SD: standard deviation.

doi:10.1371/journal.pone.0105413.t001

### Quantitative Mismatch Types: Volumetric And Estimated Mismatch

Considering all patients and all readings, mean MM_VOL_ was 66.97%±20.93% (range 12.71% to 100%) and did not differ significantly from mean MM_EST_ (62.77%±22.08%, range 0% to 100%), indicating that there was no significant systematic over- or underestimation. The two mismatch types were correlated significantly (r = 0.711, p<0.001).

Overall interreader agreement between the two readers was significantly higher for volumetric mismatch assessment as indicated by the intraclass correlation coefficient (ICC of MM_VOL_: 0.751). In contrast, overall interreader agreement of estimated mismatch was poor (ICC of MM_EST_: 0.292). In the subgroup of patients with large CBF perfusion deficits (N = 14), MM_VOL_ showed an excellent interreader agreement (ICC: 0.961). The interreader agreement of MM_EST_ (ICC: 0.415) in this subgroup was significantly higher than in the whole patient population, but still substantially lower than the agreement of MM_VOL_. In the subgroup of patients with small CBF perfusion deficits (N = 15), both MM_VOL_ (ICC: 0.482) and MM_EST_ interreader agreement (ICC: 0.131) was substantially lower than in the total sample.

Intrareader agreement for repeated assessment by the same reader was significantly higher for volumetric mismatch assessment as well (ICC of MM_VOL_: 0.843). Intrareader agreement of estimated mismatch was moderate (ICC of MM_EST_: 0.749). Intrareader agreement of both mismatch types was significantly higher in patients with large perfusion deficits. In this subgroup, MM_VOL_ (ICC: 0.942) as well as MM_EST_ (ICC: 0.919) showed very good and good intrareader agreement, respectively. Again, in the subgroup of patients with small CBF perfusion deficits, both MM_VOL_ (ICC: 0.711) and MM_EST_ intrareader agreement (ICC: 0.400) was substantially lower than in the total sample. Table 2 shows detailed data of inter- and intrareader agreement for the two quantitative types of mismatch assessment.

**Table 2:** Inter- and intrareader agreement for quantitative methods of mismatch assessment, depending on perfusion deficit volume of the patient, N = 29.

	Volumetric mismatch (MM_VOL_)	Estimated mismatch (MM_EST_)
**Interreader agreement between two different readers**		
ICC (95%CI), overall	0.75 (0.52–0.88)	0.292 (−0.10–0.63)
ICC (95%CI), large perfusion deficit*	0.96 (0.88–0.98)**	0.415 (−0.11–0.78)**
ICC (95%CI), small perfusion deficit†	0.48 (−0.07–0.81)	0.131 (−0.12–0.50)
**Intrareader agreement of repeated mismatch assessment**		
ICC (95%CI), overall	0.84 (0.57–0.94)	0.75 (0.53–0.87)
ICC (95%CI), large perfusion deficit*	0.94 (0.83–0.98)	0.92 (0.78–0.97)
ICC (95%CI), small perfusion deficit†	0.71 (0.11–0.91)	0.40 (−0.11–0.76)

* CBF perfusion deficit ≥50 mL, N = 14.

† CBF perfusion deficit <50 mL, N = 15.

** statistically significant.

ICC: intraclass correlation coefficient.

doi:10.1371/journal.pone.0105413.t002

### Semiquantitative Approach: Aspects Mismatch

Mean MM_ASPECTS_ was 2.54±2.24 (range 0 to 9), indicating that in average 2–3 ASPECTS regions were affected in CBF but not in CBV maps. Overall, there was only a slight interreader agreement of MM_ASPECTS_ as indicated by Cohen's Kappa (κ = 0.133). Interreader agreement in the subgroup of large perfusion deficits did not show satisfactory agreement either (κ = 0.205). In small perfusions deficits, interreader agreement was even lower (κ = 0.037).

Overall intrareader agreement for repeated measurements of MM_ASPECTS_ was fair (κ = 0.340). In large perfusion deficits, intrareader agreement was slightly higher (κ = 0.402), while it was lower in small perfusion deficits (κ = 0.235).

### Classification Of The Patient's Penumbra

With respect to patient classification, volumetric assessment classified 20 patients to have a favorable penumbral pattern and 9 to have a nonpenumbral pattern, (visual estimation: 24 penumbral/5 nonpenumbral, ASPECTS: 22 penumbral/7 nonpenumbral).

Both inter- and intrareader agreement was highest for volumetric classification (κ = 0.595/p = 0.002 and κ = 0.833/p<0.001, respectively), followed by visual (κ = 0.471/p = 0.013 and κ = 0.577/p = 0.002, respectively), and ASPECTS classification (κ = 0.133/p = 0.290 and κ = 0.340/p = 0.072, respectively) which failed to reach statistically significant agreement. The results of the different types of patient classification are presented in Table 3.

**Table 3:** Favorable Penumbra pattern classification (yes/no) based on volumetry, visual estimation, and ASPECTS, N = 29.

	Volumetric classification	Visual classification	ASPECTS classification
**Interreader agreement between two different readers**			
# of agreements	25	22	18
# of non-agreements	4	7	11
Cohen's Kappa	0.595	0.471	0.133
p	0.002*	0.013*	0.290
**Intrareader agreement (repeated assessment)**			
# of agreements	27	23	21
# of non-agreements	2	6	8
Cohen's Kappa	0.833	0.577	0.340
p	0.000*	0.002*	0.072

* statistically significant.

ASPECTS: Alberta Stroke Programme Early CT Score.

The values show the level of agreements within the three different methods (volumetric, visual, and ASPECTS classification).

doi:10.1371/journal.pone.0105413.t003

**Figure 2:**
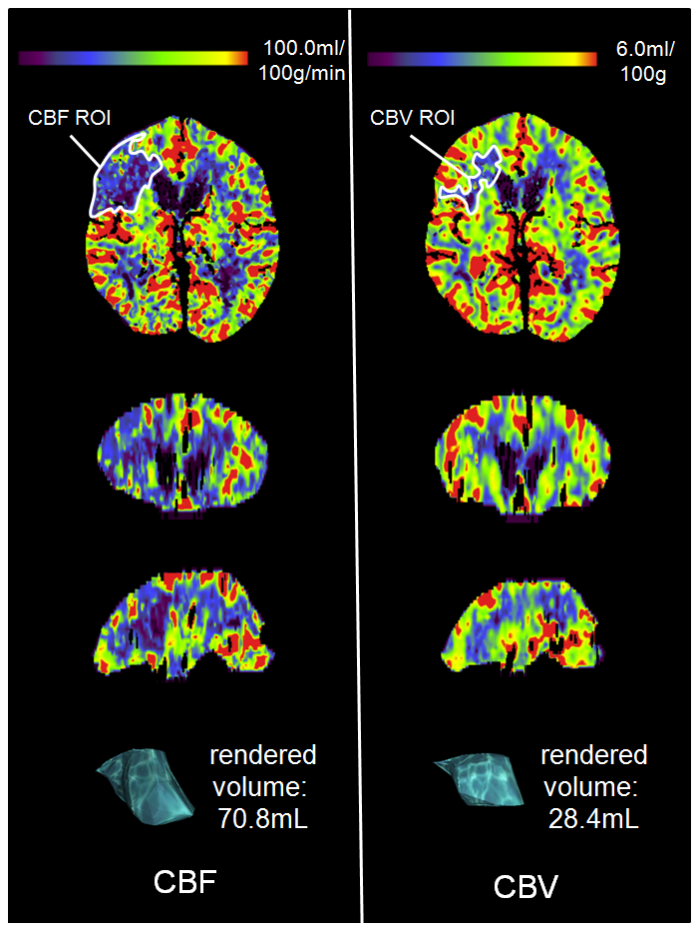
Whole brain CTP mismatch assessment in a 79 yrs old female who presented with a mild left-sided hemiparesis and facial paresis. NIHSS on admission was 2. WB-CTP was performed 185 min after symptom onset. Concerning MM_ASPECTS_, all readers rated for both CBF and CBV ASPECTS regions M1 and M4 in the right hemisphere as the only affected ones. Therefore, in none of the four readings, an ASPECTS mismatch was considered present (MM_ASPECTS_ = CBF_ASPECTS_ - CBV_ASPECTS_ = 8 - 8 = 0). However, volumetric assessment revealed an extensive mismatch of 59.9%. MM_EST_ varied from 30 to 80% (mean 57.5±26.3%). ASPECTS, Alberta Stroke Programme Early CT Score; CBF, cerebral blood flow; CBV, cerebral blood volume; NIHSS, National Institutes of Health Stroke Scale.

When comparing the classification results of both visual and ASPECTS assessment to volumetric assessment, the latter came to the same categorization in 23/29 cases (79%) when compared to visual assessment and 19/29 (66%) when compared ASPECTS assessment (Table 4). Hence, a reclassification of the penumbra pattern using volumetric assessment would have been necessary in 6/29 (21%) of the cases using visual mismatch assessment and 10/29 (33%) of the cases using ASPECTS assessment. Figure 2 shows an example of a patient in which deviant classifications result from the different mismatch assessment methods.

**Table 4:** Visual and ASPECTS penumbra classification in comparison to penumbra classification based on volumetry, N = 29.

**4b. ASPECTS categorization vs. categorization based on volumetry**		
	**Favorable according to ASPECTS†**	**Non-favorable according to ASPECTS**
Favorable according to volumetry*	16	4
Non-favorable according to volumetry	6	3

* defined by a mismatch of ≥30% in combination with an infarct core of ≤90 ml.

† defined by an ASECTS-mismatch of ≥1 point.

ASPECTS: Alberta Stroke Programme Early CT Score.

doi:10.1371/journal.pone.0105413.t004

### Time For Mismatch Assessment

Mean time for mismatch assessment differed significantly among the various mismatch assessment types (each with p<0.01). Mean time for volumetric mismatch assessment was 18:45 min (±5:11 min, range 8:48 min to 26:04 min), while it was 0:58 min (±0:17 min, range 0:25 min to 1:37 min) for visual estimation of mismatch. Semiquantitative ASPECTS mismatch assessment needed 2:48 min on average (±0:38 min, range 1:57 min to 4:06 min).

## Discussion

Cerebral blood flow-volume-mismatch is increasingly used to select patients eligible for reperfusion therapy. Therefore, reliable tools for mismatch and penumbra assessment are required. By comparing different types of mismatch assessment in whole brain CTP stroke imaging, we could show that volumetric determination of CBF-CBV-mismatch had substantially higher reliability and reproducibility than visually estimated mismatch and ASPECTS mismatch assessment.

Our findings are in line with and extend those of previous reports. The poor interreader agreement of visually estimated mismatch as it is widely performed in clinical practice is in accordance with MRI studies in which quantifying PWI-DWI mismatch visually has shown to be reproducible but not reliable among observers [7]. Post-hoc analyses of the Echoplanar Imaging Thrombolysis Evaluation Trial (EPHITHET) [17] and the Diffusion and Perfusion Imaging Evaluation for Understanding Stroke Evolution (DEFUSE) [18] datasets have shown that quantitative assessment might be superior to visual interpretation. A tool to quantitatively determine PWI-DWI mismatch was developed and has been applied to several clinical trials (RAPID) [19].

In 2-slice CT perfusion imaging of the brain, precise volumetric mismatch has been hampered by the limited brain coverage. On the other hand, whole brain CTP mismatch, similar to DWI-PWI mismatch, is three-dimensional. The poor agreement of visual mismatch estimation in our study supports the assumption that it is difficult to precisely judge the extent of 3-dimensional volumes and mismatch without the use of quantitative tools.

In a recent study, a volumetric mismatch between MTT and CBV was calculated automatically using dedicated imaging software [11]. However, in dynamic CT perfusion, thresholds for ischemic region and infarct core are still not operationally defined and universally accepted [20]. Even though automatic assessment offers the advantage of time saving, validity and accuracy have not yet been shown and most experienced CTP readers rely on their visual assessment as it preserves functional information, identification of artifacts, discrimination of grey and white matter and comparison to the contralateral side. Consequently, we deliberately avoided using rigid quantitative thresholds for the definition of infarct core and hypoperfused area as we think this approach comes closest to real-life stroke workup.

Due to its poor interreader agreement, it is debatable whether purely visual mismatch assessment is adequate in CT perfusion. This applies in particular to whole brain CTP in which 3-dimensional volumes are being compared. Our method of volumetric mismatch assessment was superior to estimated mismatch concerning reliability and reproducibility, suggesting diagnostic advantage of this approach.

ASPECTS has occasionally been suggested as a semiquantitative type of mismatch assessment [2],[13]. In stroke imaging, it is appreciated that ASPECTS provides functional weighting: functionally important regions with small volumes such as the basal ganglia are given the same weight as larger cortical regions. It could be shown that MM_ASP_ is strongly correlated to clinical outcome [21]. In our study, however, reliability and reproducibility of CTP-ASPECTS is only fair in whole brain CT perfusion. This is in contrast to a recent study of van Seeters et al. [22] who found good inter- and intraobserver agreement of ASPECTS applied to CBV and MTT maps. The discrepancy might be partly due to the fact that MM_ASPECTS_ requires two ASPECTS scores instead of one. Moreover, we had a higher proportion of patients with perfusion alterations than van Seeters et al. Nevertheless, the relatively low agreement of MM_ASPECTS_ is somewhat surprising and needs further research.

We acknowledge that a direct comparison between ASPECTS mismatch and a quantitative CBF-CBV mismatch is problematic as ASPECTS uses functional weighting, necessarily leading to a discrepancy between the two techniques. However, an ASPECTS mismatch of ≥1 has been proposed in the literature [23] as it is most compatible with the central assumption of the ASPECTS methodology – that every designated ASPECTS region is incrementally important in overall prognosis. This belief is indirectly supported by a large retrospective analysis of 825 patients in which a continuous linear relationship was observed between functional outcome and ASPECTS from 6 to 10 [24].

Apart from reliability and reproducibility results, we acknowledge that volumetric mismatch assessment was relatively time consuming. This is a major drawback in an acute emergency situation and efforts should be made to improve and to further automate the workflow of volumetric methods.

Our results must be interpreted in the context of the study design. A problem which is common to all studies in this area is that post-processing methods differ between manufacturers and tissue thresholds that distinguish penumbra from irreversibly damaged tissue have been derived in relatively small patient groups with widely varying criteria for tissue viability.

As a second limitation relates to fact that the measurement of agreement by means of ICCs can be misleading as correlation analyses are weighted towards the extremes of a spectrum. Hence, even if correlations are poor for individual patients, ICCs can be good within a large group of subjects. Although our data indicate that the quantitative approach is superior, we additionally performed a binary categorization and an agreement analysis using Kappa-values.

Third, as the aim of our study was to determine reliability and reproducibility, we used follow-up MRI only to confirm unilateral MCA stroke and not to correlate CT perfusion deficits with final infarction volumes. Future studies on diagnostic accuracy may further investigate the predictive value of 3-dimensional mismatch. Finally, our study is limited to 29 subjects. Future studies might show whether our results can be confirmed in larger samples.

In conclusion, we could show that volumetric mismatch assessment in recently introduced WB-CTP is more reliable and reproducible than visual mismatch assessment as it is widely performed in clinical practice. CTP-ASPECTS preserves functional information but results in a different patient classification than the other approaches. Volumetry might provide improvement of therapeutic decision making in ischemic stroke patients, especially in cases with large perfusion deficits. In small perfusion deficits, contrariwise, relative mismatch approaches should be used cautiously.
